# Associations Between Age-Related Changes in Positive Affect and Physical Functioning: The Role of Education

**DOI:** 10.1093/geroni/igab046.2199

**Published:** 2021-12-17

**Authors:** Maria Blöchl, Timo von Oertzen, Ute Kunzmann

**Affiliations:** 1 Leipzig University, Leipzig University, Sachsen, Germany; 2 Universität der Bundeswehr, Neubiberg, Bayern, Germany; 3 Leipzig University, Leipzig, Sachsen, Germany

## Abstract

Increasing research points to the relevance of educational attainment for positive emotional experiences and physical functioning across adulthood. However, little is known about how age-related developments in positive affect and physical functioning differ by educational attainment. This study used longitudinal data of 10,893 individuals (60–80 years) from the Health and Retirement Study to examine whether educational attainment moderates trajectories of positive affect and physical functioning and their interrelations over 12 years. Initial results from multiple-group bivariate growth models revealed that individuals with less formal education have lower positive affect and poorer physical functioning at baseline. There was, however, no evidence that longitudinal changes in positive affect, longitudinal changes in physical health, and coupled changes between both variables varied with educational attainment. These initial findings suggest that lower educational attainment is primarily related to lower levels of positive affect and physical functioning, but not to greater age-related declines or their interrelations.

